# Supplementary material for: MetaRibo-Seq measures translation in microbiomes
Source: Nat Commun. 2020 Jun 29;11:3268. doi: 10.1038/s41467-020-17081-z (PMC7324362; doi:10.1038/s41467-020-17081-z)
Supplement: Supplementary file 10 — Supplementary Data 7 [file 41467_2020_17081_MOESM10_ESM.zip › File2/Confidence_High_Taxonomy/358861_out.krona.html]

Javascript must be enabled to view this page.

members
magnitude
magnitudeUnassigned
count
unassigned
taxon
rank

358861\_out

8

8
superkingdom
2

phylum
8
1239

186801
class
4

186802
4
order

31979
1
family

1
genus
1485


SRS024132\_contig\_number\_50244
1262845
1
species

3
family
186803

1898203
2
species

SRS018836\_contig\_number\_26575SRS077024\_contig\_number\_14605

genus
1
841


SRS015065\_contig\_number\_14470
species
1
166486


SRS019808\_contig\_number\_20844SRS054352\_contig\_number\_19541SRS098881\_contig\_number\_3178
1897036
species
3

2292897
1
species

SRS075821\_contig\_number\_25005
